# PTEN-related risk classification models for predicting prognosis and immunotherapy response of hepatocellular carcinoma

**DOI:** 10.1007/s12672-023-00743-x

**Published:** 2023-07-20

**Authors:** Lu Cao, Xiaoqian Ma, Juan Zhang, Cejun Yang, Pengfei Rong, Wei Wang

**Affiliations:** 1 Department of Radiology, The Third Xiangya Hospital, Central South University, Changsha, 410005 China; 2 The Institute for Cell Transplantation and Gene Therapy, Central South University, Changsha, 410005 China; 3 Postdoctoral Research Station of Special Medicine, The Third Xiangya Hospital, Central South University, Changsha, China

**Keywords:** PTEN, Tumor microenvironment, Mutation, Hepatocellular carcinoma, Prognostic classification model, Immunotherapy response

## Abstract

**Introduction:**

PTEN often mutates in tumors, and its manipulation is suggested to be used in the development of preclinical tools in cancer research. This study aims to explore the biological impact of gene expression related to PTEN mutations and to develop a prognostic classification model based on the heterogeneity of PTEN expression, and to explore its sensitivity as an indicator of prognosis and molecular and biologic features in hepatocellular carcinoma (HCC).

**Material and methods:**

RNA-seq data and mutation data of the LIHC cohort sample downloaded from The Cancer Genome Atlas (TCGA). The HCC samples were grouped according to the mean expression of PTEN, and the tumor microenvironment (TME) was evaluated by ESTIMATE and ssGSEA. The prognostic classification model related to PTEN were constructed by COX and LASSO regression analysis of differentially expressed genes (DEGs) between PTEN-high and -low expressed group.

**Results:**

The expression of PTEN was affected by copy number variation (CNV) and negatively correlated with immune score, IFNγ score and immune cell infiltration. 1281 DEGs were detected between PTEN-high and PTEN-low expressed group, 8 of the DEGs were finally filtered for developing a prognosis classification model. This model showed better prognostic value than other clinicopathological parameters, and the prediction accuracy of prognosis and ICB treatment for immunotherapy cohorts was better than that of TIDE model.

**Conclusions:**

This study demonstrated the effect of CNV on PTEN expression and the negative immune correlation of PTEN, and constructed a classification model related to the expression of PTEN, which was of guiding significance for evaluating prognostic results of HCC patients and ICB treatment response of cancer immunotherapy cohorts.

**Supplementary Information:**

The online version contains supplementary material available at 10.1007/s12672-023-00743-x.

## Introduction

Hepatocellular carcinoma (HCC) ranks the second highest cause to cancer-related deaths in the world, with major causes such as hepatitis C virus (HCV) infection, non-alcoholic steatohepatitis, chronic alcohol intake, hepatitis B virus (HBV) infection, [1, 2]. The current prognosis for HCC patients is extremely poor, with 5-year overall survival rates ranging from 10 to 20% [3]. Variable parameters such as serum AFP level, des-gamma-carboxy prothrombin level, tumor size and number, margin status, major vessel invasion, tumor stage, Edmonson-Steiner grade, Child–Pugh score, portal hypertension and cirrhosis, are regarded as clinical prognosticators of HCC, hinging on the treatment modalities and underling liver diseases [4]. However, traditional biomarkers are not ideal for risk stratification, prognosis, and treatment response prediction of HCC development in high-risk populations [5]. There are indications that that the genetic changes of pretumor hepatocytes and the gradual accumulation of mutations in the same cells are the causes of malignant transformation leading to the development of HCC [6]. Genomic studies have identified the prospect of HCC molecular changes; however, only about 25% of HCC have potentially operable mutations that have not yet been translated into clinical practice [7, 8]. Understanding the influence of mutations in HCC development may open the way for the identification of personalized biomarkers.

Phosphatase and tensin homologue (PTEN), which a tumor suppressor gene located in the long arm of chromosome 10 at position 23.3 that often manifests mutation in cancer, could suppress tumorigenesis via a variety of mechanisms, for example, subcellular localization and protein–protein interaction, phosphatase-dependent and independent activities, and it regulates many cellular functions including DNA repair and cell movement, cell growth, proliferation, and survival [9, 10]. The mutation and deletion of PTEN are related to a series of clinical results of the tumor. PTEN gene mutation in advanced cervical cancer is associated with tumor progression and adverse outcome after radiotherapy [11]. PTEN loss is identified as a clinically related genetic change that drives the molecular and histopathological heterogeneity of lung tumors caused by Rb1/Trp53 mutations [12]. Mutations, decreased promoter activity and decreased expression in PTEN were also reported in patients with HCC [13], their dysregulation has a critical pathogenic role in HCC development. Future studied are encouraged to probe into the regulation of PTEN expression level and its downstream signaling to facilitate the design of therapeutic strategies for treating HCC as well as some other cancers [14].

The current study used bioinformatics analysis to evaluate the biological effects of PTEN mutation-regulated expression, including cancer stem cell (CSC), carcinogenic signal pathways and tumor microenvironment (TME). We were also committed to developing a prognostic classification model based on the heterogeneity of PTEN expression to explore its sensitivity as an indicator of HCC prognosis and immunotherapy response, which may be helpful for HCC prognosis and post-treatment monitoring.

## Materials and methods

### Collection and curation of HCC data

Using GDC API to view and download HCC sample clinicopathological parameters and data in The Cancer Genome Atlas (TCGA), including RNA sequencing data, copy number variation (CNV), and somatic mutation data. The HCC sample RNA-seq data and prognosis information of GSE14520 dataset were obtained from NCBI. The HCC sample data with missing prognostic information in TCGA and GSE14520 data sets were not in the sample we considered. Finally, a total of 355 HCC samples and 50 paracancer samples from TCGA were shortlisted for analysis in this study. 221 tumor samples and 220 paracancer samples from the GSE14520 dataset were also analyzable data for this study.

### Differential expression analysis based on PTEN

The expression of PTEN in HCC and paracancer samples was analyzed in TCGA database and GEO database respectively, and the difference of PTEN expression in the two types of tissues was compared by T test. PTEN mutation and CNV type were used as the classification basis respectively, and PTEN expression of samples was used as the variable to analyze the differences between groups with genomic variation.

### Correlation analysis between PTEN and stemness index

The PTEN pathway is known to control normal stem cell maintenance, self-renewal, and migration. PTEN loss can lead to the emergence and proliferation of CSC clones [15]. Stemness index based on mRNA expression (mRNAsi) obtained by one-class logistic regression machine-learning algorithm (OCLR) was an indicator of cancer stemness [16]. The mRNAsi of the TCGA-LIHC cohort sample was calculated using this algorithm, which was then used for Pearson correlation analysis with PTEN, and the degree of correlation was judged by the value of Pearson correlation coefficients.

### Analysis of immune abundance based on PTEN expression

According to the mean value of PTEN expression in TCGA-LIHC, the samples above the mean value were set as PTEN high expressed group, and the samples below the mean value were set as PTEN low expressed group. The immune score of HCC based on PTEN expression grouping was quantized according to specific signatures related to the infiltration of immune cells in tumor tissues using ESTIMATE algorithm [17]. IFN-γ is also a major cytokine involved in immune regulation. The IFN-γ score was obtained by ssGSEA of “GOBP_RESPONSE_TO_INTERFERON_GAMMA gene set” downloading from the MsigDB database (http://www.gsea-msigdb.org/gsea/index.jsp), as one of the immune indicators evaluated in this study. Meanwhile, using the strategy proposed by Charoentongm [18], 28 subpopulations of tumor-infiltrating lymphocytes (TILs) including major types associated with adaptive immunity for HCC samples was analyzed. The enrichment degree of 29 gene signatures in HCC samples was evaluated according to the global method covering cellular and functional properties of TME created by Bagaev et al. [19].

### Gene set enrichment analysis (GSEA)

Information on 10 molecular mechanisms including NRF2, Hippo, cell cycle, Notch, β-catenin/WNT, Myc, RTK-RAS, PI-3-Kinase/Akt, TGF β signaling, p53 was obtained from a report on “Oncogenic signaling pathways” [20]. Pearson correlation analysis took the GSEA score and PTEN expression of these pathways as variables to measure the correlation. The pathway viability based on PTEN expression was quantified in the GSEA website (http://www.gsea-msigdb.org/gsea/index.jsp), in which the selected gene set was “h.all.v7.5.1.symbols.gmt” and the threshold of significance was defined as p.adj < 0.25.

### Construction and verification of prognostic classification model

The gene expression profiles of PTEN high expression group and PTEN low expression group were uploaded to “Limma” to calculate the difference of gene expression profile between the two groups. The differentially expressed genes (DEGs) should meet the requirements of FDR < 0.05 and |log2FC| > log2(1.5). The univariate COX regression model was constructed by using the survival data of DEGs, and the genes with p < 0.001 were uploaded to “glmnet” package. The least absolute shrinkage and selection operator (LASSO) regression model was constructed by tenfold cross validation, which selected the combination of the least genes related to HCC survival to produce the final prognostic classification model:

$$Risk\;score = \mathop \sum \limits_{i = 1}^{n} \left( {\beta_{i} \times Exp_{i} } \right)$$

Here n represented the gene number of the model, i was the gene, beta was the coefficient, and Exp was the expression level. Kaplan–Meier curves were employed to describe the association between risk score and overall survival (OS). Receiver operating characteristic (ROC) curve used the area under the curve (AUC) as an index to judge the specificity and sensitivity of the prognostic classification model for predicting OS.

### Evaluation of prognostic classification models in immune checkpoint blocking (ICB) therapy genomes

We collected genomic and clinical information from two ICB treatment datasets: the IMvigor210 dataset treated with atezolizumab [21] and the GSE78220 dataset treated with pembrolizumab [22]. Tumor Immune dysfunction and ejection (TIDE) is often used to evaluate responses to ICB treatment [23]. TIDE and prognostic classification models were introduced into the two ICB treatment data sets to calculate risk score and evaluate OS. The prediction accuracy of the two models for immunotherapy response was also evaluated and compared by AUC.

### Establishment of a nomogram

Univariate and multivariate Cox regression analysis were used to identify independent prognostic variables in age, gender, tumor stage and grade and risk score, which were integrated into a nomogram by the “RMS” package. The calibration chart was used to compare the 1-year, 3-year and 5-year OS predicted by nomogram and actually observed. The accuracy of nomogram in predicting OS was evaluated by decision curve analysis (DCA) and ROC curve.

### Statistical analysis

All the statistical analysis processes were completed by R software. The differences of variables in accordance with normal distribution were calculated by Student’s t-test, and the differences of non-normal distribution variables were evaluated by Wilcoxon rank sum test. Kaplan–Meier curve and ROC curve were drawn by “survival” package and “timeROC” package, respectively. The P value represents the degree of statistical difference, and the significance level of the analysis without mentioning the threshold was set to 0.05.

## Results

### Effects of PTEN mutation in HCC on expression and biological pathway

**Fig. 1:**
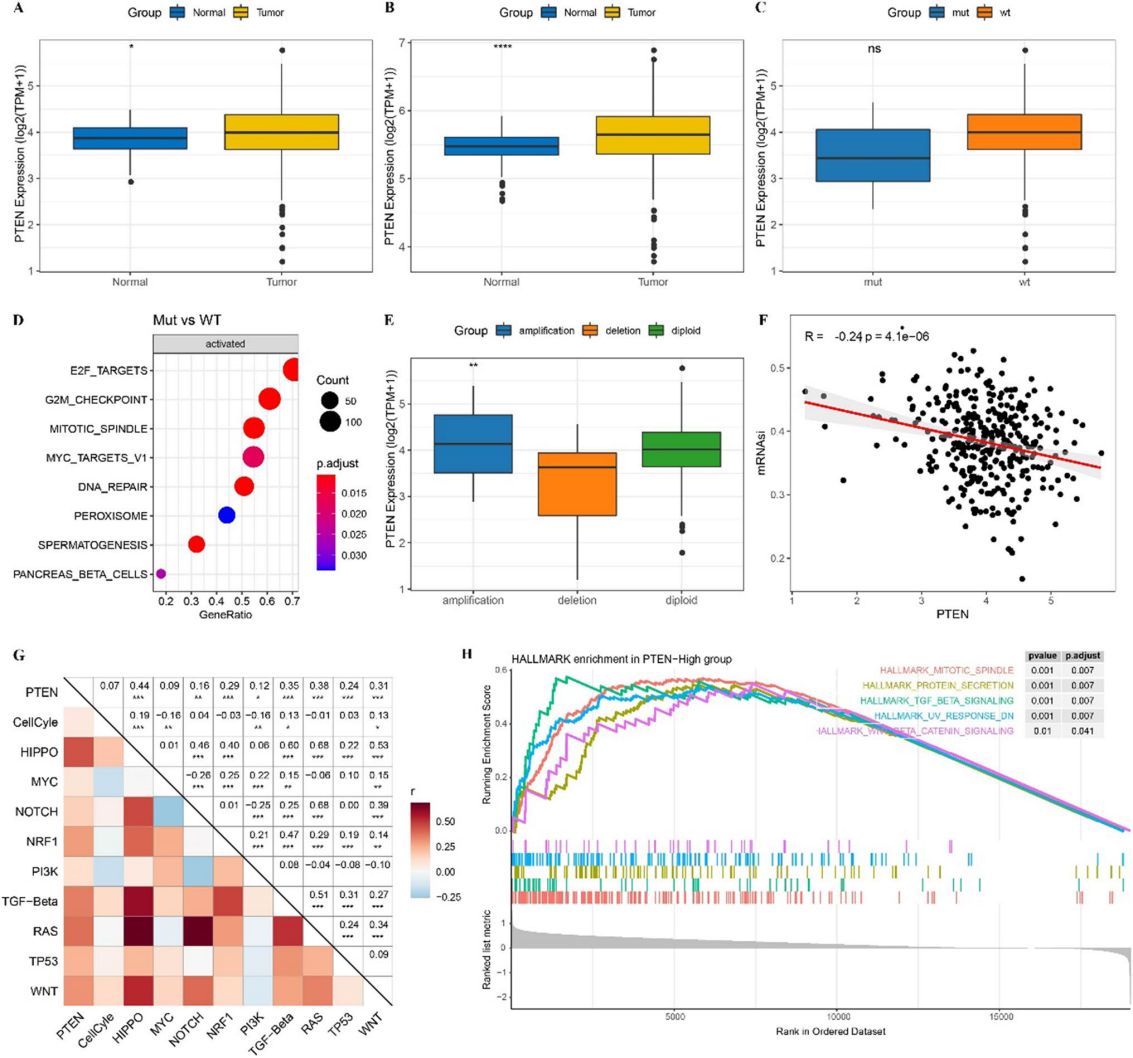
Effects of PTEN mutation in HCC on expression and biological pathway. **A** The differential expression of PTEN in HCC tumor tissues and paracancer tissues in the TCGA-LIHC cohort. **B** The differential expression of PTEN between tumor tissue and paracancer tissue in GSE14520 data set. **C** The expression of PTEN in PTEN mutant and wild type samples of TCGA-LIHC dataset. **D** The significant enrichment pathway of PTEN mutant samples relative to wild type samples. **E** PTEN expression of different CNV types in TCGA-LIHC cohort. **F** Pearson correlation analysis between PTEN expression and mRNAsi. **G** Pearson correlation analysis between PTEN expression and 10 carcinogenic signaling pathways. **H** GSEA for patients with high expressed PTEN and low expressed PTEN. *Ns* no significant difference, *p < 0.05, **p < 0.01, ***p < 0.001, ****p < 0.0001

Before exploring the role of PTEN in HCC, the differential expression of PTEN in HCC tumor samples and paracancer samples was analyzed. In TCGA-LIHC cohort and GSE14520 cohort, PTEN was significantly overexpressed in HCC samples as compared with that in paracancer samples (Fig. 1A, B). The mutation information of PTEN gene was sorted out by using the maf data of TCGA, and 8 samples with PTEN mutation and 334 samples without PTEN mutation were found. We observed that the expression of PTEN in PTEN mutant samples was lower than that in wild type samples, but there was no significant difference in PTEN expression between the two types of samples (Fig. 1C). In comparison to the wild-type samples, mitotic spindle, MYC targets, DNA repair, G2M checkpoint, E2F targets, and other signal pathways were significantly enriched in PTEN mutant samples (Fig. 1D). In the aspect of CNV, the PTEN CNV type of 2 samples in TCGA-LIHC cohort was copy number amplification, the CNV type of PTEN in 22 samples was copy number deletion, and the CNV of PTEN does not exist in 329 samples. Significant differences in PTEN expression were detected among the three types of samples, and PTEN expression was significantly inhibited in samples with copy number deletion compared with samples with copy number amplification and CNV without PTEN (Fig. 1E). The results here indicated that CNV affects the expression of PTEN. Therefore, we went on to analyze the biological effects of PTEN expression. The correlation of cancer stem cell (CSC) of PTEN was explored by Pearson correlation analysis between PTEN expression and mRNAsi. We detected a significant negative correlation between PTEN and mRNAsi (Fig. 1F). The expression of PTEN was also positively correlated with carcinogenic signal pathways including PI3K, TGF- β, NOTCH, TP53 and WNT, HIPPO, NRF1, and RAS (Fig. 1G). Patients with HCC were divided into groups according to the average amount of PTEN expression in the TCGA-LIHC cohort. Twelve pathways were identified that were significantly more highly enriched in PTEN-High group than PTEN-low group, including mitotic spindle, protein secretion, TGF-β signaling, UV response, WNT β catenin signaling, etc. (Fig. 1H).

### The influence of PTEN on TME

**Fig. 2:**
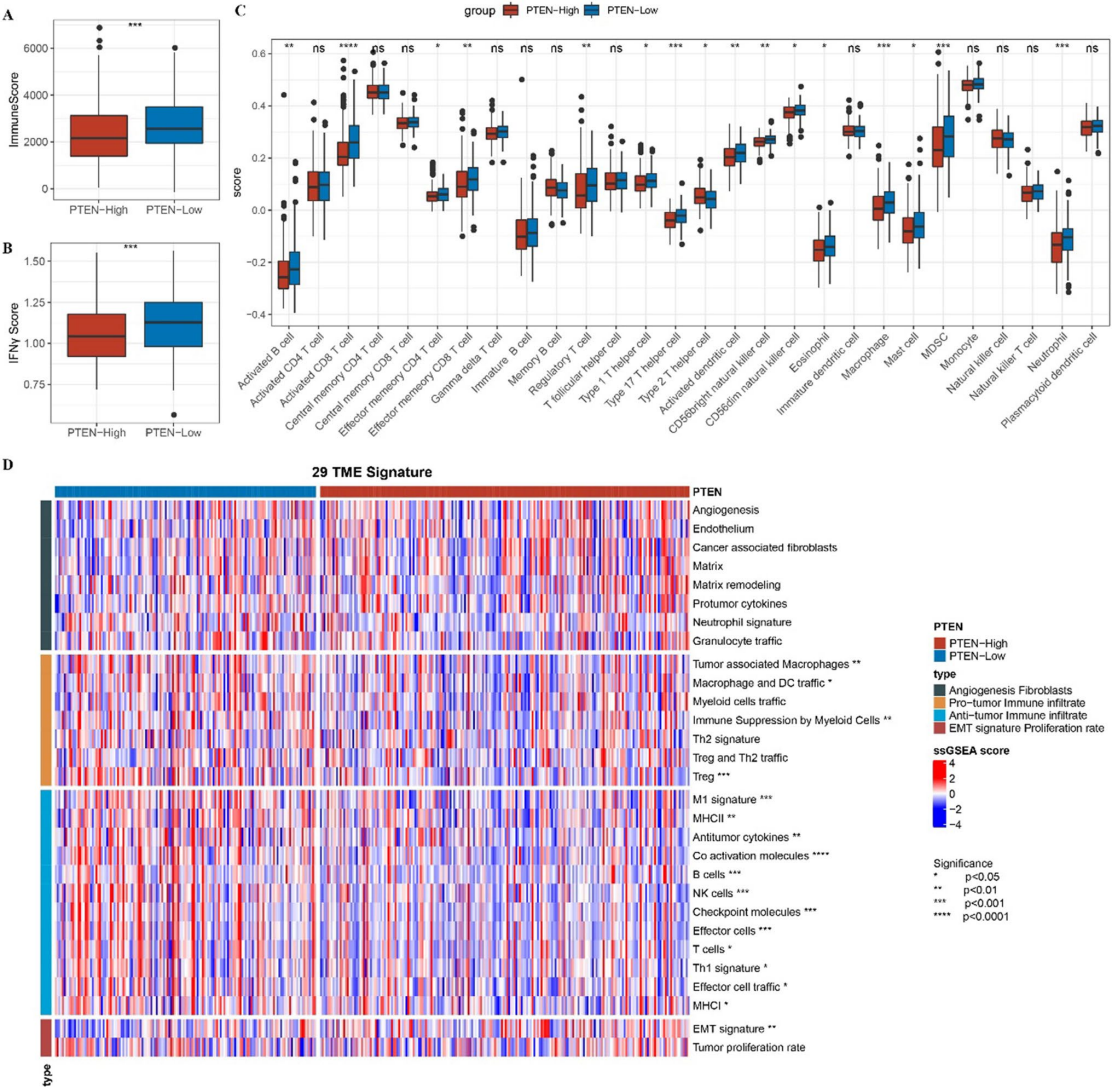
The influence of PTEN on TME. **A** The difference of immune score between the cases with high expression of PTEN and low expression of PTEN. **B** IFN γ score in patients with high expression of PTEN and low expression of PTEN. **C** The enrichment scores of 28 TILs in cases with high expression of PTEN and low expression of PTEN. **D** The heat map shows the distribution of ssGSEA calculated based on the gene signatures of 29 indicative TME in the cases with high expression of PTEN and low expression of PTEN. *Ns* no significant difference, *p < 0.05, **p < 0.01, ***p < 0.001, ****p < 0.0001

Patients with HCC were divided into groups according to the average amount of PTEN expression in the TCGA-LIHC cohort. The abundance of immune score, immune cells and 29 gene signatures representing the main functional components, immunity, matrix and other cell groups of tumor were evaluated in two different PTEN expression groups. Compared with cases with low PTEN expression, the immune score and IFN-γ score were significantly reduced in cases with high PTEN expression (Fig. 2A, B). We detected a significant difference in the abundance of 16 TILs between the cases with high expressed PTEN and the cases with low expressed PTEN. Compared with the patients with low expression of PTEN, patients with high expression of PTEN had significantly elevated abundance of type 2T helper cell and significantly decreased abundance of activated B cell, macrophage, effector memory CD4 T cell, regulatory T cell, effector memory CD8 T cell, activated CD8 T cell, type 17T helper cell, type 1T helper cell, myeloid-derived suppressor cell (MDSC), CD56 bright natural killer cell, activated dendritic cell, eosinophil, neutrophil, mast cell, CD56dim natural killer cell (Fig. 2C). The results calculated by gene signatures indicating 29 TME components showed that the enrichment of 17 TME-related components was significantly lower in cases with high expression of PTEN than in cases with low expression of PTEN (Fig. 2D).

### Construction and validation of prognostic classification model based on 8 genes

**Fig. 3:**
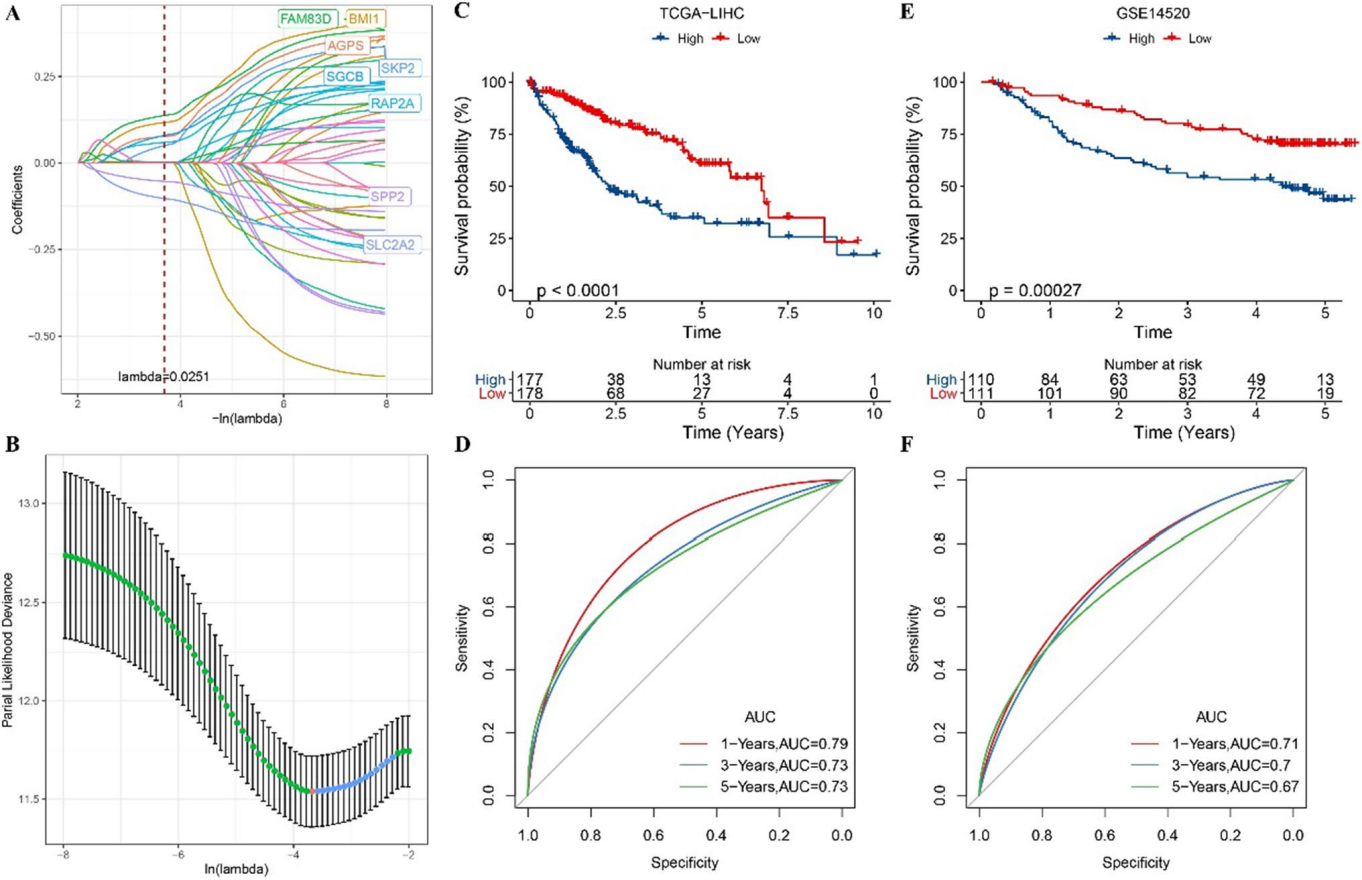
Construction and validation of prognostic classification model based on 8 genes. **A** The LASSO coefficient profiles. **B** Cross-validation for lambda screening in the LASSO regression model based on minimum criteria for OS. **C** The OS of samples based on prognostic classification model in TCGA-LIHC cohort. **D** ROC curve of prognostic classification model in TCGA-LIHC cohort. **E** Prognostic classification model for OS prediction of samples in the GSE14520 cohort. **F** The specificity and sensitivity of prognostic classification model in TCGA-LIHC cohort

Prognosis of TCGA-LIHC cohort cannot be significantly stratified based on PTEN expression (Figure S1), we screened DEGs between groups with high PTEN expression and those with low PTEN expression to construct a prognosis classification model. 1281 DEGs were detected between PTEN high expression group and low expression group in TCGA-LIHC cohort, 1249 were up-regulated DEGs and 32 were down-regulated DEGs (Figure S2A). These DEGs were significantly related to microRNA in cancer, pathways in cancer, signaling pathways regulating pluripotency of stem cells, focal adhesion and hepatocellular carcinoma (Figure S2B). From the results of Gene Ontology (GO) analysis, it was observed that biological process (BP) and cellular component (CC) and molecular function (MF), which were significantly related to DEGs, were all involved in epithelial–mesenchymal transformation (EMT) (Figure S2C). Univariate Cox regression analysis of 1281 DEGs identified 48 genes that were statistically significant for prognosis. LASSO regression analysis selected the combination of 8 genes related to HCC survival to produce the final prognostic classification model (Fig. 3A, B): risk score = 0.136 × BMI1 + 0.088 × AGPS + 0.076 × RAP2A + 0.162 × FAM83D + 0.085 × SKP2 + (− 0.122 × SLC2A2) + 0.109 × SGCB + (− 0.064 × SPP2). Based on the median of risk score in the TCGA-LIHC cohort (training set) and the GSE14520 cohort (validation set), we summarized high-risk cases and low-risk cases. In terms of OS, low-risk cases always had a significant advantage over low-risk cases at any point in time (Fig. 3C, E). The 1-year AUC of the prognosis classification model in the TCGA-LIHC cohort and the GSE14520 cohort were 0.79 and 0.71, respectively; the 3-year AUC were 0.73 and 0.7, respectively; And the 5-year AUC were 0.73 and 0.67, respectively (Fig. 3D, F).

### Predictive effect of prognostic classification model on prognosis and immunotherapy response in ICB treatment cohort

**Fig. 4:**
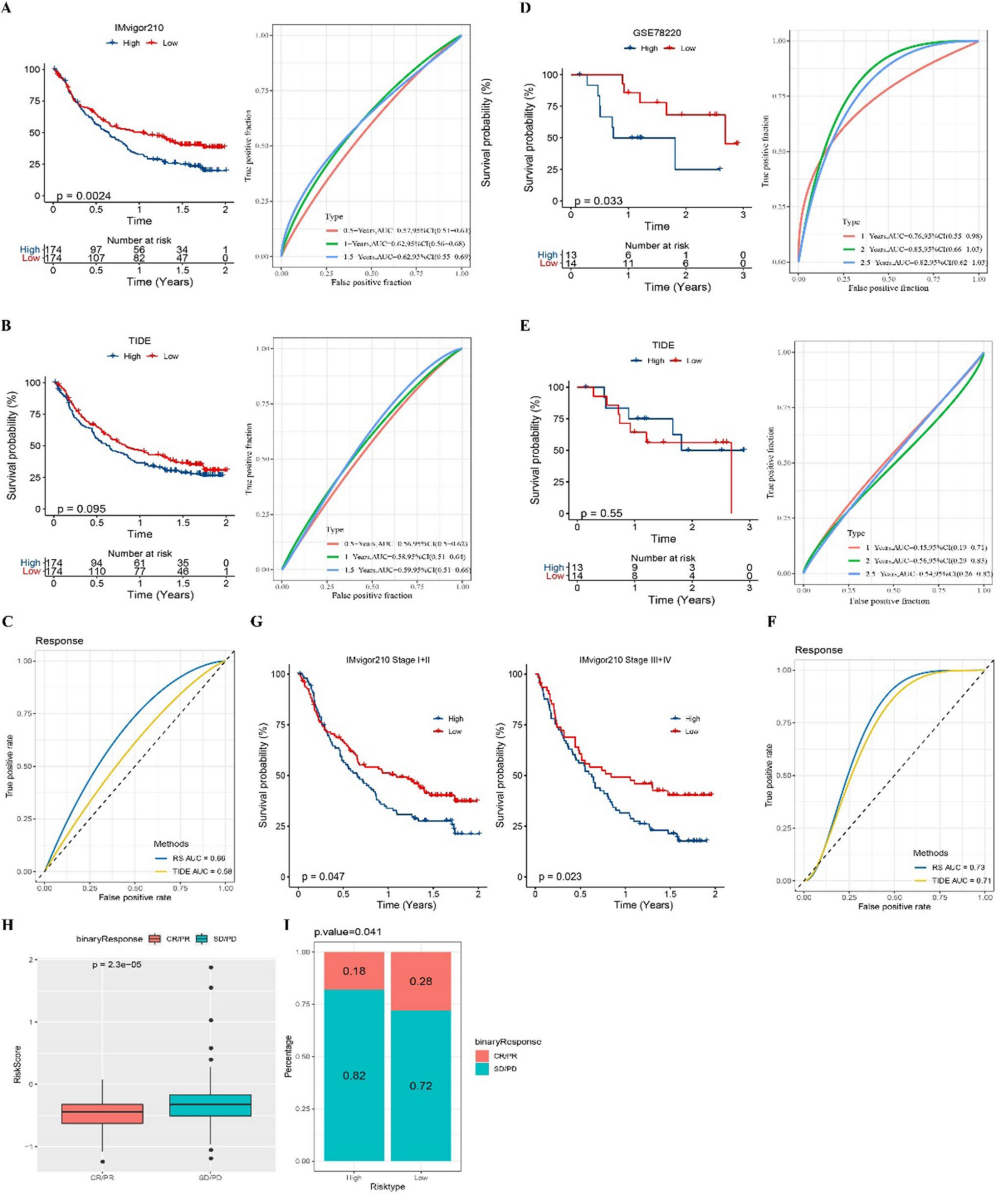
Predictive effect of prognostic classification model on prognosis and immunotherapy response in ICB treatment cohort. **A** The prognostic classification model predicted Kaplan–Meier curve and ROC curve in the IMvigor210 cohort. **B** TIDE predicts the Kaplan–Meier curve and ROC curve of OS in the IMvigor210 cohort. **C** Prognostic classification model and TIDE were used to predict the ROC curve of ICB treatment response in the IMvigor210 cohort. **D** Kaplan–Meier curve and ROC curve of OS were evaluated by prognostic classification model in the GSE78220 cohort. **E** TIDE evaluated the Kaplan–Meier curve and ROC curve of prognosis in the GSE78220 cohort. **F** Prognostic classification model and TIDE predict AUC of ICB therapy response in the GSE78220 cohort. **G** Prognostic classification model for survival prediction of different stage in IMvigor210 cohort. **H** risk score in SD/PD cases and CR/PR cases. **I** Response rates of the two risk groups in the IMvigor210 cohort to ICB treatment

The prognostic classification model and TIDE were introduced into two ICB treatment cohorts to evaluate the OS of the samples. The prognostic classification model was statistically significant in predicting the OS of IMvigor210 cohort samples, and its AUC reached 0.62 at 1 year and 1.5 years (Fig. 4A). TIDE had no statistical significance in predicting the OS of IMvigor210 cohort samples, and the AUC of predicting 1-year and 1.5-year OS did not reach 0.6 (Fig. 4B). In terms of the accuracy of predicting the treatment response of ICB, the efficiency of prognostic classification model was higher than that of TIDE (0.68 vs 0.58) (Fig. 4C). The prognostic classification model also significantly distinguished the OS of patients in different risk groups in the GSE78220 cohort, and the AUC of 1 year, 2 years and 2.5 years were 0.76, 0.85 and 0.82, respectively (Fig. 4D). There was no significant correlation between TIDE score and OS calculated by TIDE in the GSE78220 cohort, and the AUC in 1 year, 2 years and 3 years was much lower than that in the prognostic classification model (Fig. 4E). In this cohort, the prognostic classification model also had more potential to predict ICB than TIDE (0.73 vs 0.71) (Fig. 4F). Moreover, no matter which clinical stage case is in, the prognostic classification model could significantly distinguish different survival outcomes (Fig. 4G). In the IMvigor210 cohort, 348 patients showed varying degrees of response to PD-L1 receptor blockers, including stable disease (SD) and progressive disease (PD), partial response (PR), complete response (CR). The level of risk score in SD/PD cases was significantly higher than that in CR/PR cases (Fig. 4H). Among the two risk groups, the proportion of CR/PR in the low-risk group was significantly higher than that in the high-risk group, so it was judged that the response of low-risk cases to ICB was better than that of high-risk cases (Fig. 4I).

### Construction and verification of nomogram based on prognostic classification model

**Fig. 5:**
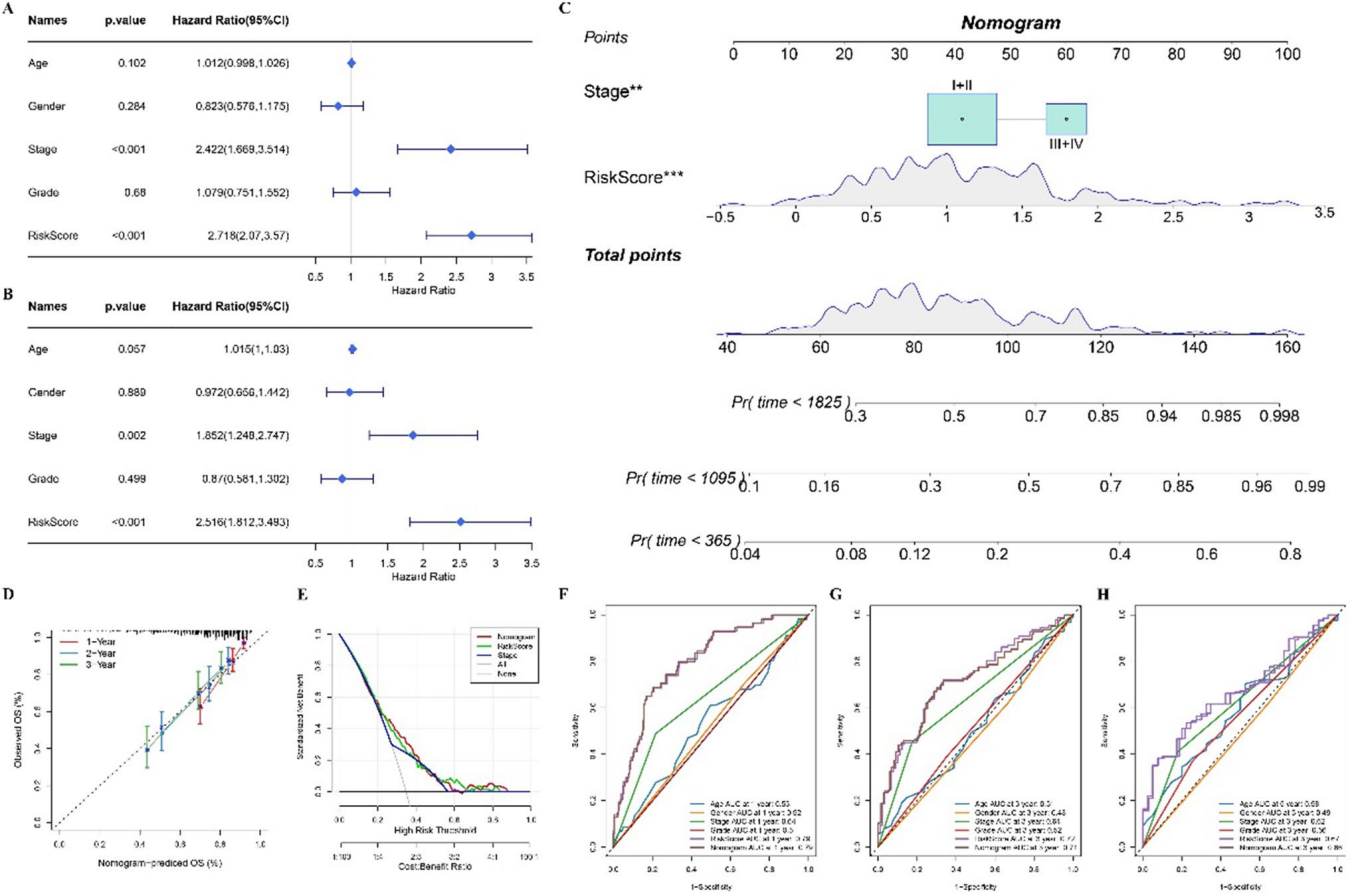
Construction and verification of nomogram based on prognostic classification model. **A** Univariate Cox regression analysis of risk score and clinical variables age, gender, clinical stage and grade. **B** Multivariate Cox regression analysis shows the independent variables related to the prognosis of HCC. **C** Nomogram built from clinical stage and risk score. **D** The calibration curve showed the degree of coincidence between the 1-year, 2-year and 3-year OS predicted by nomogram and the 45° dashed line. **E** The net benefit of nomogram and risk score and clinical stage in predicting the prognosis of HCC. **F**–**H** ROC curve shows the 1-year, 3-year and 5-year AUC of OS predicted by nomogram, risk score and clinical variables age, gender, clinical stage and grade

By univariate Cox regression analysis of clinical variables and risk score, the significant variable clinical stage and risk score was introduced into the multivariate COX regression model, and the significant variables in the univariate model were also significant in the multivariate model, indicating that clinical stage and risk score was an independent variable related to the prognosis of HCC (Fig. 5A, B). Thus, clinical and risk score were combined into a nomogram (Fig. 5C). The calibration curve showed that the 2-year and 3-year OS of HCC predicted by nomogram almost coincided with the 45° dotted line, indicating that nomogram could accurately predict the prognosis of HCC (Fig. 5D). The net benefit of nomogram and risk score in predicting the prognosis of HCC was observed in the DCA curve (Fig. 5E). In the 1-year, 3-year and 5-year OS forecasts for HCC, the AUC of nomogram and risk score was significantly higher than that of age, gender and clinical stage and grade (Fig. 5F–H).

### Clinical characteristics, mutation and biological correlation of prognostic classification model

**Fig. 6:**
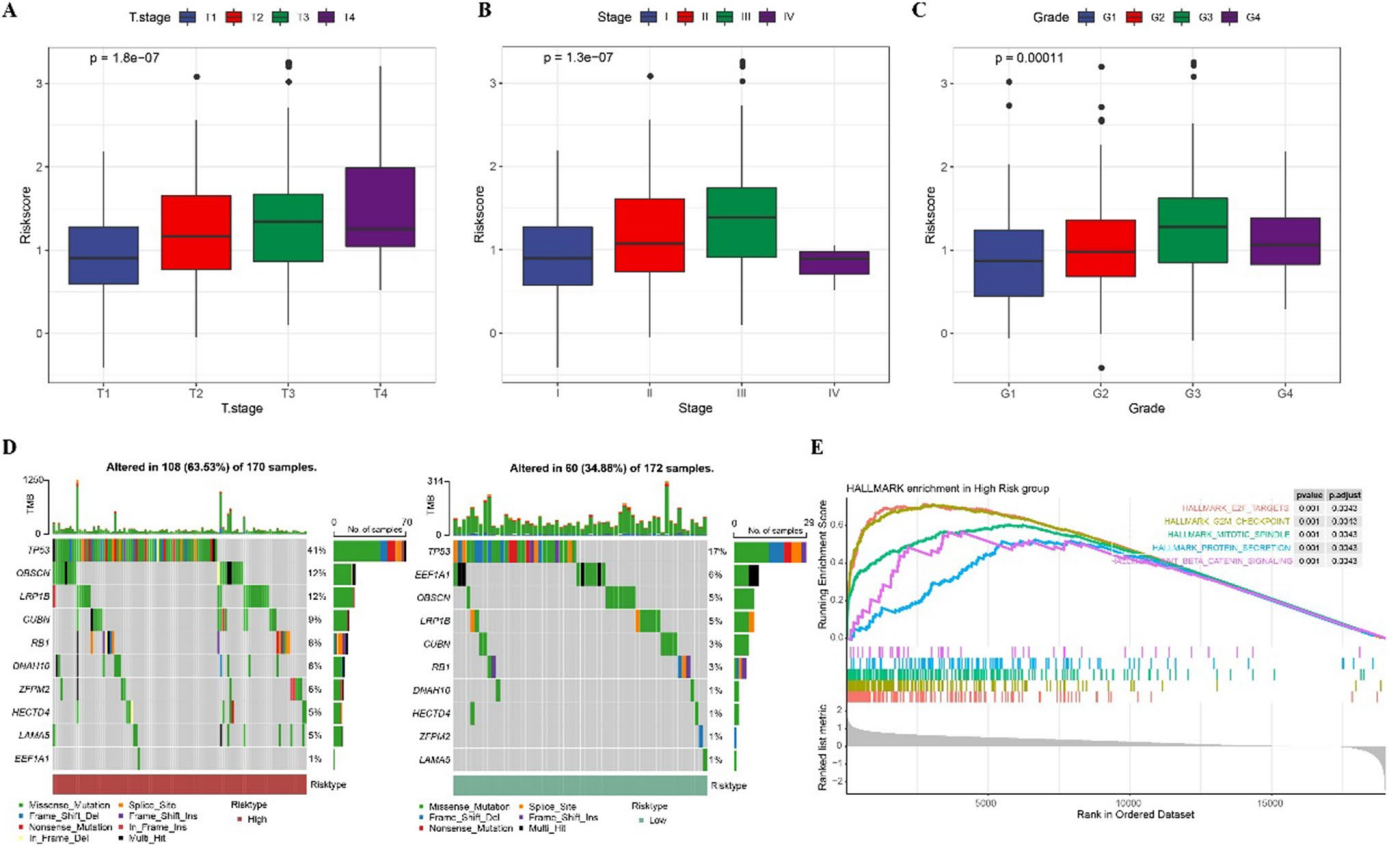
Clinical characteristics, mutation and biological correlation of prognostic classification model. **A** Risk score of HCC cases with different T stage. **B** Risk score in HCC cases with different clinical stage. **C** The difference of risk score in HCC cases with different grade. **D** The mutation patterns of the 10 genes with the highest mutation rates in high-risk and low-risk cases. **E** Enrichment differences in biological pathways between high-risk and low-risk cases identified by GSEA

The clinical correlation of prognostic classification model was explored. We found that risk score had different levels in different T stages, different clinical stages and different grades groups. The risk score of T2–T4 was significantly higher than that of T1 (Fig. 6A). Similarly, the sample at the later clinical stage exhibited a higher risk score than the sample at the earlier clinical stage (Fig. 6B). High-grade HCC cases showed higher risk score than low-grade HCC cases (Fig. 6C). Compared with low-risk cases, the mutation rate of high-risk cases increased significantly, especially TP53, with a mutation rate of 41% in high-risk cases and 17% in low-risk cases. The mutation pattern of this gene was also different in different risk cases (Fig. 6D). Compared with low-risk cases, the activity of 18 pathways in high-risk cases was significantly increased, among which the pathways with the greatest difference in activity between risk groups were those regulating the cell cycle, such as mitotic spindle, E2F targets and G2M checkpoint (Fig. 6E). These results indicate that the prognostic classification model is beneficial to the identification of different mutation characteristics and biological functions in HCC cases.

## Discussion

Advances in understanding the molecular pathogenesis of HCC have led to the identification of key driving mutations. PTEN is the second most common mutation gene in HCC [24]. The most frequently observed mechanisms of PTEN mutation are single gene copy deletion and gene silencing [25]. At the molecular level, the change of PTEN expression/activity is related to several cellular defects in liver and non-hepatocytes, thus promoting carcinogenesis [26]. Functionally, PTEN regulates genomic stability, cancer immunogenicity, immune cell infiltration and different cancer immune responses [27]. In mouse experiments, the lack of hepatocyte specific PTEN led to the occurrence of HCC in mice [28, 29]. PTEN manipulation is suggested to be used in the development of preclinical tools in cancer research [30]. In this study, we explored the differences of PTEN expression and biological function between PTEN mutant samples and wild type samples, analyzed the correlation between PTEN expression and HCC prognosis and TME, and established a prognostic classification model based on PTEN manipulation.

We analyzed the expression of PTEN between PTEN wild type samples and mutant samples, and detected that the gene expression of the samples without PTEN mutation was higher than that of the samples with PTEN mutation, but there was no significant difference. We speculated that this might be the reason for the small number of samples, because we only found PTEN mutation in 8 samples out of 342 samples. It is speculated that this may be the reason why the number of samples is too small, because we only found PTEN mutations in 8 samples. As previously reported, PTEN optimizes genomic integrity by regulating key processes both inside and outside the cell cycle, and harmful cascades of cell cycle defects are triggered after PTEN mutation or inactivation, such as the accumulation of DNA damage and the inactivation of cell cycle checkpoints [31]. Echoing previous studies, we detected differences in several regulatory pathways of the cell cycle process, such as E2F, G2M checkpoint, mitotic spindle, and DNA repair, between PTEN mutant samples and wild-type samples. Some evidence reveals that PTEN deletion promotes CSC compartments in solid and hematological malignancies [10]. Our results also confirmed the negative correlation between PTEN expression and mRNAsi, indicating the negative correlation trend between PTEN expression and CSC. PTEN deletion induces TME remodeling, which is related to immunosuppressive infrastructure in TME [32]. In this study, according to the average expression of PTEN, we achieved HCC grouping based on PTEN, confirmed the effect of PTEN on TME, and the high expression of PTEN was significantly negatively correlated with the abundance of immune cell infiltration and immune activity.

Considering that PTEN has been proved to be of guiding significance in predicting the prognosis of liver cancer [33], and in view of the heterogeneity of TME in the grouping of HCC cases expressed by PTEN, this study focused on the DEGs between the patients with high expression of PTEN and low expression of PTEN, and designed a multi-gene classification model to guide the prognosis prediction of HCC. The model was linked with clinicopathological features including T stage, clinical stage and grade, and could accurately predict the prognosis of HCC. A previous study confirmed that PTEN deletion was associated with poorer outcomes of PD-1 inhibitor-based immunotherapy in preclinical models of melanoma [34]. In this study, the PTEN difference-identified model we constructed showed higher accuracy than TIDE in distinguishing patient outcomes and ICB response in an immunotherapy cohort of metastatic urothelial cancer and metastatic melanoma.

In the prognostic classification model, BMI1 is a member of the polycomb protein family, acts as an oncogene in the carcinogenesis of hepatocellular carcinoma in an independent manner of INK4a/ARF loci, and has been proved to be a qualified therapeutic target for HCC [35]. According to Benjamin et al., AGPS maintains ether lipids, controls the cellular utilization of fatty acids, and helps to produce signal lipids necessary to promote cancer invasiveness [36]. RAP2A shows a strong ability to distinguish tumor from normal tissue. Its expression is correlated with progressive tumor grade, TP53 and CTNNB1 mutation status, and becomes an independent prognostic marker of HCC patients [37]. FAM83D has been reported to be overexpressed in HCC and leads to a poor prognosis [38]. Many genes drive the malignant biological behavior of HCC by regulating SKP2, such as EAG1 [39] and DNAJC5 [40]. A report based on bioinformatics analysis found that SLC2A2 was independently associated with the OS of patients with HCC [41]. Although these genes had different effects on HCC, the role of their prognostic classification model in HCC has been reported for the first time.

In conclusion, we found the mutation and overexpression of PTEN in HCC and the negative correlation between PTEN and TME. We also designed a PTEN-related classification model, which showed ideal accuracy in judging the prognosis and immunotherapy response of HCC, and related to the clinicopathological features including T stage, clinical stage and grade. To sum up, our study provided a model for predicting the prognosis and immunotherapy response of HCC, which might be of great significance for personalized treatment of HCC.

## Supplementary Information

Below is the link to the electronic supplementary material.

Supplementary file 1—Figure S1. Prognosis of HCC stratified based on PTEN expression (PDF 193 KB)

Supplementary file 2—Figure S2. DEGs and its biological function between PTEN high expression group and low expression group. A: DEGs between PTEN high expression group versus low expression group in TCGA-LIHC cohort, the blue dot on the left side represents the down-regulated DEGs, the red dot on the right indicates the up-regulated DEGs, and the gray dot is the gene that does not show significant difference between the PTEN high expression group and the low expression group. B: Kyoto Encyclopedia of Genes and Genomes (KEGG) analysis for DEGs between PTEN high expression group and low expression group in TCGA-LIHC cohort. C: DEGs-enriched GO terms between PTEN high expression group and low expression group of TCGA-LIHC cohort. (PDF 11183 KB)

## Data Availability

The data used to support the findings of this study are included within the article.

## Abbreviations

PTEN: Phosphatase and tensin homologue
HCC: Hepatocellular carcinoma
TCGA: The Cancer Genome Atlas
GEO: Gene Expression Omnibus
TME: Tumor microenvironment
DEG: Differentially expressed gene
GSEA: Gene set enrichment analysis
SsGSEA: Single-sample gene set enrichment analysis
CNV: Copy number variation
HBV: Hepatitis B virus
HCV: Hepatitis C virus
CSC: Cancer stem cell
TIL: Tumor-infiltrating lymphocyte
OS: Overall survival
ROC: Receiver operating characteristic
AUC: Area under the curve
TIDE: Tumor Immune dysfunction and ejection
DCA: Decision curve analysis
GO: Gene Ontology
BP: Biological process
CC: Cellular component
MF: Molecular function
EMT: Epithelial–mesenchymal transformation
LASSO: Least absolute shrinkage and selection operator
