# Supplementary figures and images for: PTEN-related risk classification models for predicting prognosis and immunotherapy response of hepatocellular carcinoma
Source: Discov Oncol. 2023 Jul 20;14:134. doi: 10.1007/s12672-023-00743-x (PMC10361452; doi:10.1007/s12672-023-00743-x)

## TCGA-LIHC

Survival probability (%)

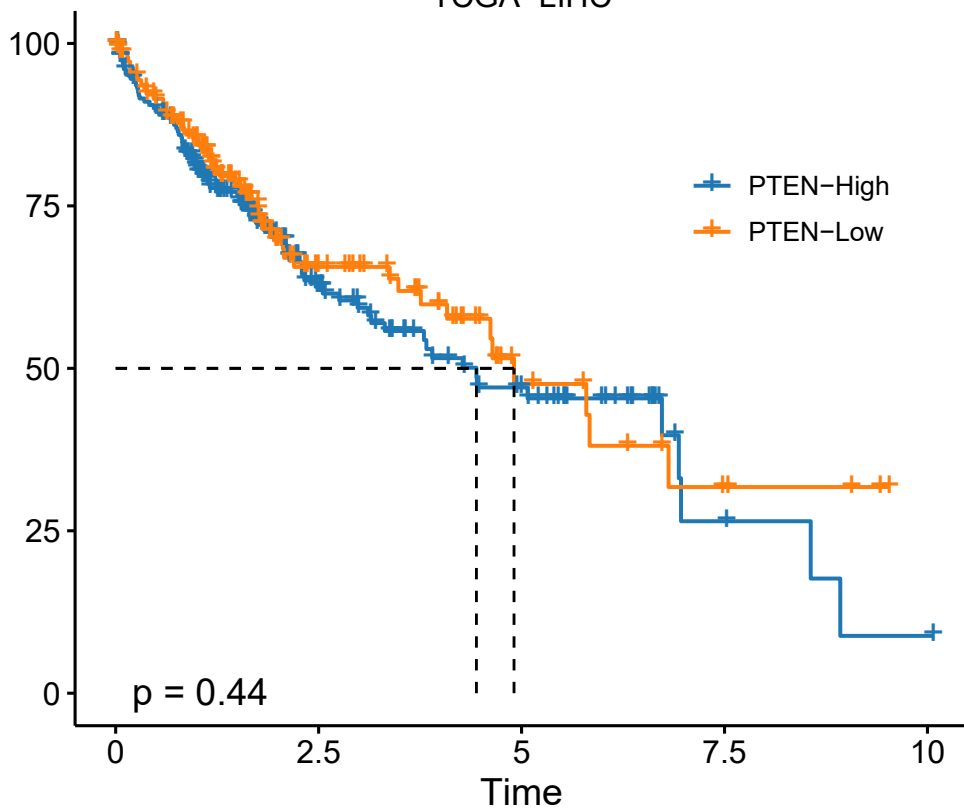 $p = 0.44$ 

Time

Number at risk

|           | 0   | 2.5 | 5  | 7.5 | 10 |
|-----------|-----|-----|----|-----|----|
| PTEN-High | 208 | 63  | 28 | 4   | 1  |
| PTEN-Low  | 147 | 43  | 12 | 4   | 0  |

Supplement: Supplementary file 1 — Figure S1. Prognosis of HCC stratified based on PTEN expression (PDF 193 KB) [file 12672_2023_743_MOESM1_ESM.pdf]

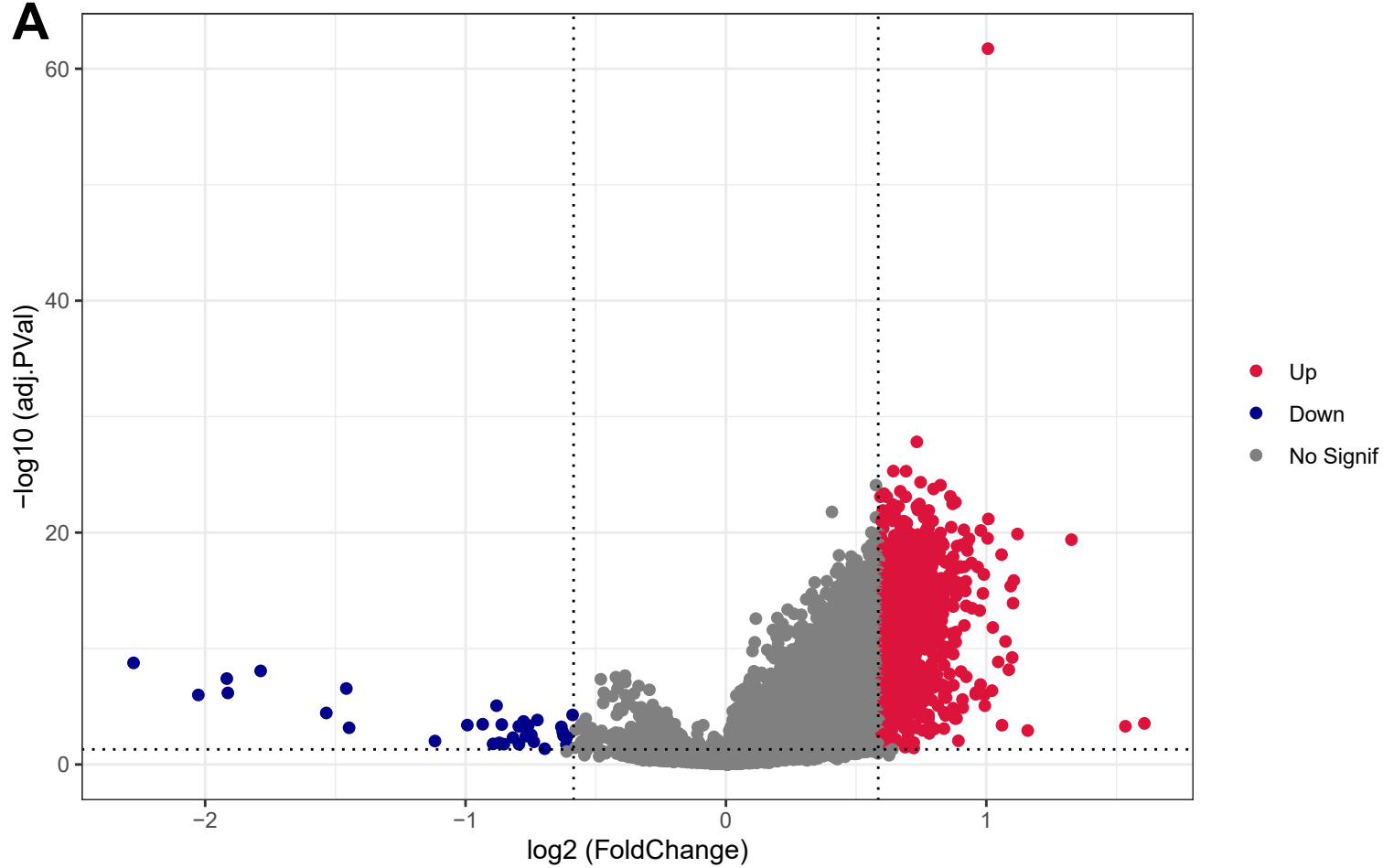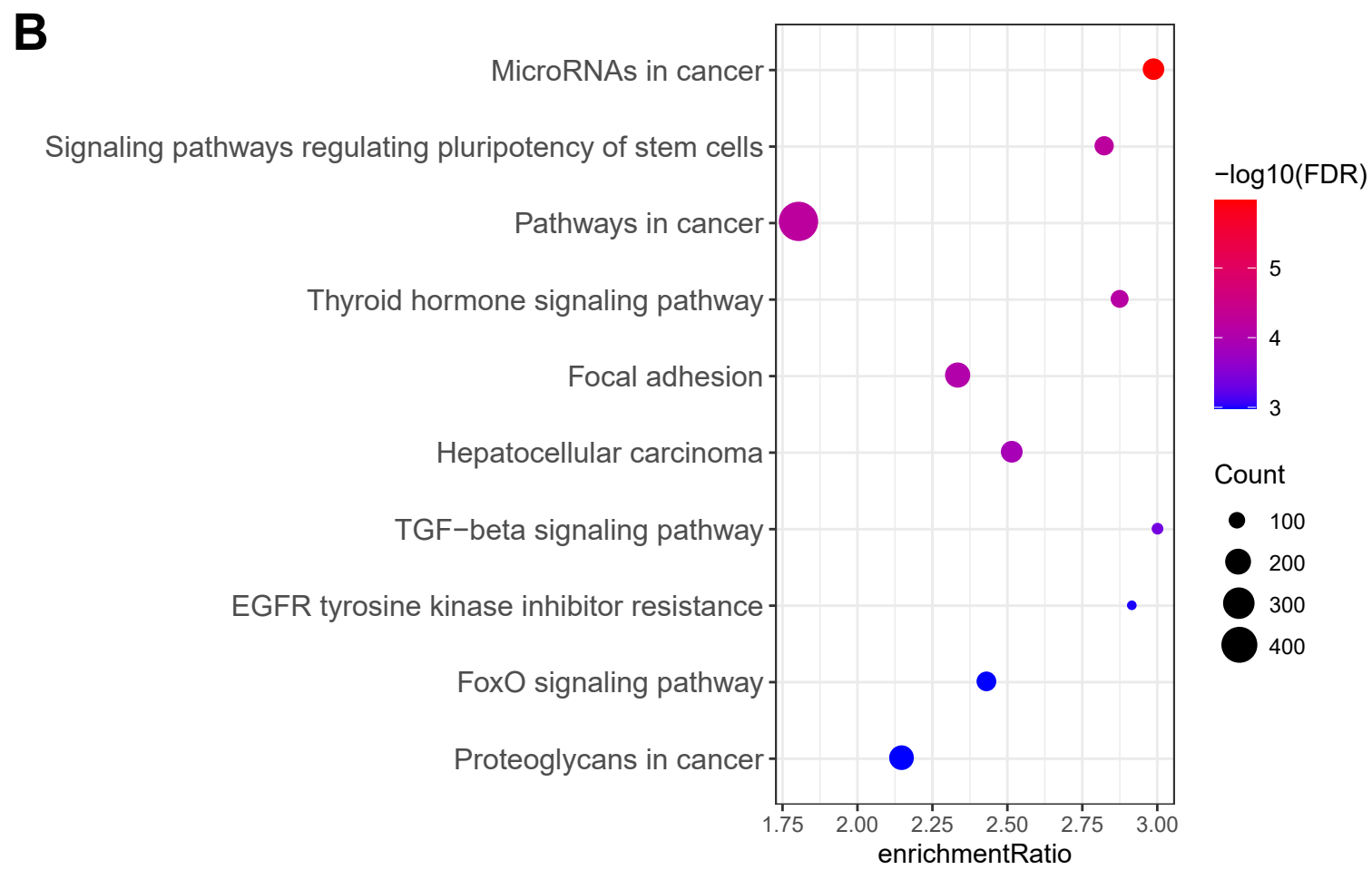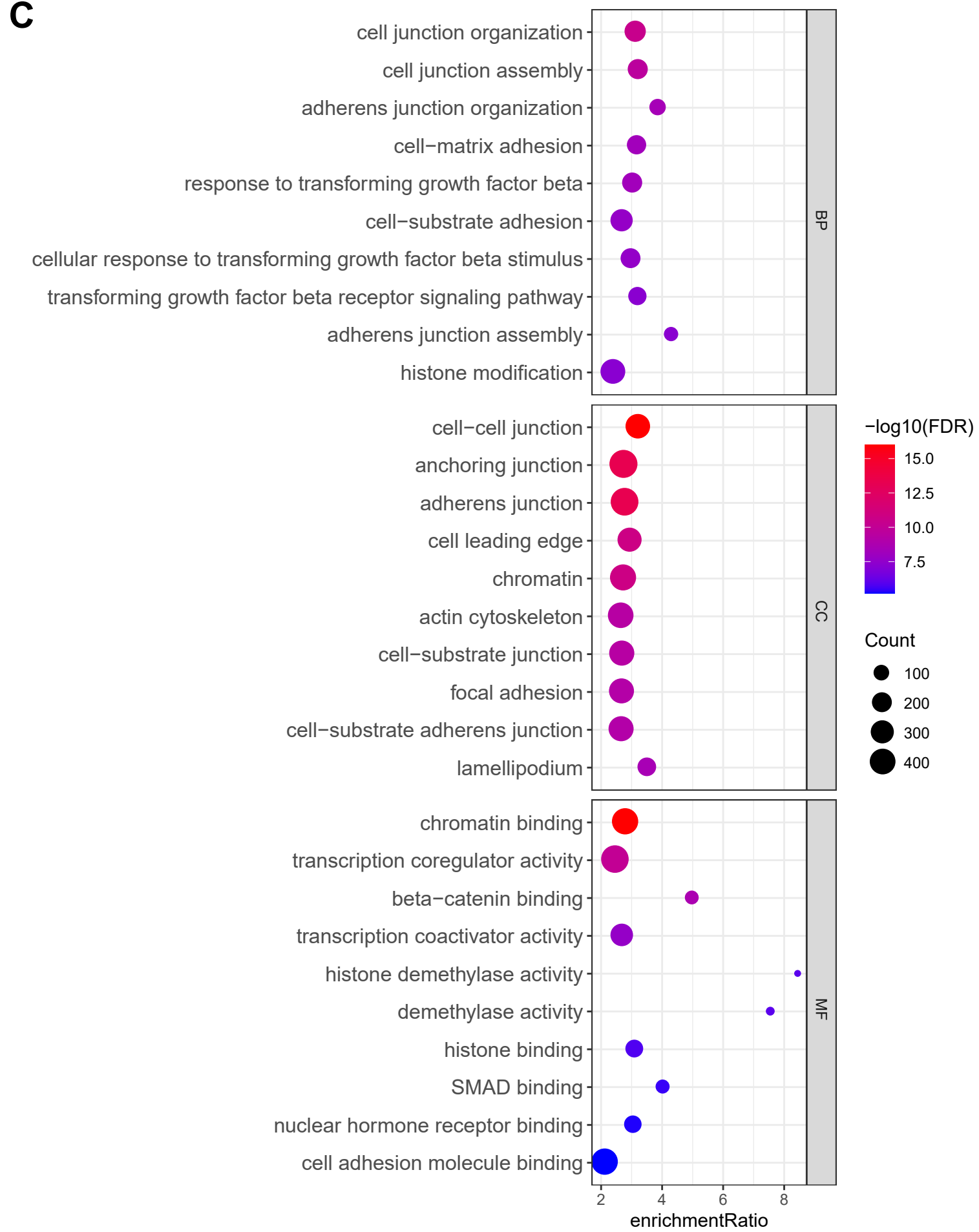

Supplement: Supplementary file 2 — Figure S2. DEGs and its biological function between PTEN high expression group and low expression group. A: DEGs between PTEN high expression group versus low expression group in TCGA-LIHC cohort, the blue dot on the left side represents the down-regulated DEGs, the red dot on the right indicates the up-regulated DEGs, and the gray dot is the gene that does not show significant difference between the PTEN high expression group and the low expression group. B: Kyoto Encyclopedia of Genes and Genomes (KEGG) analysis for DEGs between PTEN high expression group and low expression group in TCGA-LIHC cohort. C: DEGs-enriched GO terms between PTEN high expression group and low expression group of TCGA-LIHC cohort. (PDF 11183 KB) [file 12672_2023_743_MOESM2_ESM.pdf]
